# Pathological tumor long‐to‐short axis ratio as a prognostic factor in patients with thymic epithelial tumors

**DOI:** 10.1111/1759-7714.14582

**Published:** 2022-07-21

**Authors:** Dong Tian, Haruhiko Shiiya, Masaaki Sato, Aya Shinozaki‐Ushiku, Hao‐Ji Yan, Jun Nakajima

**Affiliations:** ^1^ Department of Thoracic Surgery The University of Tokyo Graduate School of Medicine Tokyo Japan; ^2^ Academician (Expert) Workstation Affiliated Hospital of North Sichuan Medical College Nanchong China; ^3^ Department of Thoracic Surgery, West China Hospital Sichuan University Chengdu China; ^4^ Department of Cardiovascular and Thoracic Surgery Hokkaido University Graduate School of Medicine Sapporo Japan; ^5^ Department of Pathology The University of Tokyo Graduate School of Medicine Tokyo Japan; ^6^ School of Medical Image North Sichuan Medical College Nanchong China

**Keywords:** long‐to‐short axis ratio, thymic epithelial tumors, survival, prognostic factor, prediction model

## Abstract

**Background:**

Thymic epithelial tumors (TETs) exhibit irregular shapes reflective of the heterogeneity in tumor growth and invasive properties. We aimed to identify the prognostic value of the pathological tumor long‐to‐short axis (L/S) ratio in TETs.

**Methods:**

A retrospective study was performed on patients with TETs who underwent extended thymectomy between January 1999 and December 2019 in our institute. Patients were divided into two groups according to the threshold of the L/S ratio. Overall survival (OS) and progression‐free survival (PFS) were evaluated by Kaplan‐Meier analysis. The independent prognostic factors of TETs were identified by multivariate analysis. The performance of prediction models for the above survival outcomes with and without the L/S ratio was evaluated using an integrated time‐dependent area under the curve (iAUC).

**Results:**

Eligible patients were divided into two groups based on higher (*n* = 42) and lower (*n* = 94) L/S ratios according to a threshold value of 1.39. A significant difference was found between the two groups only in disease progression (*p* = 0.001). Poorer survival outcomes were found from Kaplan‐Meier curves in the higher L/S ratio group (*p* < 0.05). In the multivariable analysis, the L/S ratio showed significant effects on OS and PFS (*p* < 0.05). The performance of models with the L/S ratio was better than that without the L/S ratio in predicting survival outcomes.

**Conclusions:**

The pathological tumor L/S ratio is an independent prognostic factor for OS and PFS in patients with TETs, and an L/S ratio >1.39 is associated with worse survival outcomes.

## Graphical abstract

Thymic epithelial tumors (TETs) exhibit irregular shapes reflective of the heterogeneity in tumor growth and invasive properties. We retrospectively reviewed the patients with TETs who underwent extended thymectomy between January 1999 and December 2019 in our institute and found that: (i) An L/S ratio > 1.39 was significantly correlated with a greater incidence of disease progression and poorer survival outcomes. (ii) In addition to the TNM stage and WHO classification, the L/S ratio was the only independent prognostic factor for OS and PFS. (iii) The prediction models with the L/S ratio achieved better performance than those without the L/S ratio for 5‐ to 15‐years survival outcomes.

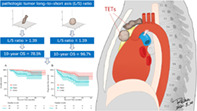

## INTRODUCTION

Thymic epithelial tumors (TETs) are rare tumors occurring in approximately 1.3–3.2/10^6^ individuals.^1^, ^2^ They represent the most common disease entity of the anterior mediastinum and are associated with favorable survival.^3^, ^4^, ^5^ Many cases with TETs are incidentally detected by unrelated diagnostics or during the diagnostic workup of paraneoplastic syndromes, such as myasthenia gravis (MG).^6^ The indolent clinical course and rarity of TETs have limited the available evidence for identifying prognostic factors, which is critical for determining appropriate treatment and surveillance strategies. Previous publications have proposed many independent prognostic factors for TETs, such as tumor size, tumor stage, and World Health Organization (WHO) histological classification.^7^, ^8^, ^9^ However, most of these criteria are still considered controversial.

Due to the irregular shape of TETs, the tumor size may not accurately reflect their heterogeneity in morphological characteristics.^10^ However, the tumor shape, often defined as round, oval, and irregular/lobulated according to the tumor long‐to‐short (L/S) ratio, is an anatomical feature of TETs that may provide more accurate information than tumor size. Previous literature on parathyroid carcinomas and lung cancer has demonstrated the significance of tumor shape or the L/S ratio in predicting postoperative survival or tumor staging.^11^, ^12^, ^13^ Qu and colleagues^14^ reported a close relationship between tumor shape and the Masaoka stage in thymoma. However, no study has documented the relationship between the pathological tumor L/S ratio and survival outcomes in TETs.

The 2021 WHO Classification refers to the TETs into thymoma (type A, AB, B1, B2, B3) and thymic carcinoma. We reviewed patients with TETs, including thymoma and thymic carcinoma, because it is difficult to differentiate the TET subtypes in terms of the morphology or histopathology.^15^ In this current study, we sought to explore the threshold of the L/S ratio and to investigate the correlation between the L/S ratio and survival outcomes. Moreover, we identified independent prognostic factors and further evaluated the prognostic value of the L/S ratio, which may guide clinicians in making appropriate decisions regarding postoperative treatment and follow‐up surveillance for patients with TETs. To the best of our knowledge, this is the first report to propose the L/S ratio as a new prognostic factor for TETs.

## METHODS

### Study population

The Institutional Review Board of The University of Tokyo Hospital approved this study before we conducted current study (no. 2406). The requirement for individual patient consent was waived due to the retrospective nature of this study. In our institute, routinely extended thymectomy has been adopted as the standard treatment regardless of the tumor stage or the presence of myasthenia gravis since 1976 to control postoperative MG and recurrence.^8^, ^9^, ^16^, ^17^ In the procedure of extended thymectomy, the tumor, thymus gland, and surrounding tissues of the anterior mediastinum were resected. In addition, we resected the neighboring tissues and organs involved by the invasive TETs. In cases with pleural dissemination, an additional pleuropneumonectomy or pleurectomy was performed based on need.^16^, ^17^ All patients with TETs at our institute between January 1999 and December 2019 were reviewed, and a total of 151 consecutive patients with TETs were initially collected. The selection criteria included: (a) patient age ≥ 20 years; (b) histologically confirmed TETs; and (c) pathological information available for restaging according to the eighth edition of the tumor, node, metastasis (TNM) staging classification by the American Joint Committee on Cancer (AJCC)/the Union for International Cancer Control (UICC).^10^ We excluded one patient who was younger than 20 years of age, three with neuroendocrine tumors, three who received preoperative induction therapy, four with ambiguous WHO classifications, and four who did not have detailed information regarding the pathological tumor size; thus, 136 patients were ultimately enrolled for further analysis. The flow chart for patient recruitment and exclusion is shown in Figure E1. These patients had also been included in our previously reported patient population.^8^

### Definitions and data collection

The data extracted from each patient's medical record included age, sex, pathological tumor longest‐ and shortest‐axis, surgical approach, WHO classification, adjuvant therapy, TNM stage, MG, surgical radicality, operation date, date of the last follow‐up, and patient status in terms of death, recurrence or disease progression. The WHO classification was based on the 2021 WHO classification system.^15^ The TNM staging records were reviewed and restaged according to the eighth edition TNM staging system.^10^ As we previously described, the surgical radicality of R0 resection represented a negative margin in microscopy.^8^ Patients with non‐R0 resection or more invasive tumors would consider adjuvant therapy, including radiotherapy and chemotherapy. After surgery, follow‐up was performed every 3–6 months, with the last follow‐up visit occurring in April 2021.

Fresh pathological specimens were analyzed before formalin fixation to prevent potential changes in tumor shape and size.^11^, ^18^ The tumors were recorded in three dimensions by pathologists according to consistent measurements recorded as “a × b × c”. Briefly, the longest tumor axis (labeled as “a”) was first measured. Then, the next longest axis (labeled as “b”) perpendicular to “a” was scaled on another dimension. Finally, the third dimension (label “c”) was defined as the length of the longest axis perpendicular to the “a” and “b” plane. Therefore, the L/S ratio was calculated as the ratio of the longest and shortest axis among labels “a”, “b”, or “c”. The definitions of survival outcomes followed the standard outcome measures for thymic malignancies of the International Thymic Malignancy Interest Group (ITMIG).^19^ In brief, overall survival (OS) was defined as the time interval between the date of operation and the date of death. Progression‐free survival (PFS) and disease‐free survival (DFS) were calculated from the date of resection of the primary tumors to the date of the diagnosis of disease progression (whole population) and recurrence (patients who underwent R0 resection), respectively.

### Statistical analysis

We divided the patients into higher and lower L/S ratio groups based on the threshold of the L/S ratio for predicting 10‐year OS, which was optimized by maximizing sensitivity plus specificity from the time‐dependent receiver operating characteristic (tROC) curve as we previously reported.^9^ The tROC curve analysis extends the standard cross‐sectional ROC curve into the longitudinal setting using survival analysis techniques. It assesses the discriminatory power of continuous variables for time‐dependent disease outcomes.^20^ Differences in patient clinicopathological characteristics between higher and lower L/S ratios were evaluated using Pearson's chi‐square test or the continuity‐adjusted chi‐square test and independent‐sample *t*‐test. The Kaplan‐Meier method was used to analyze OS, PFS, and DFS, and the log‐rank test was used to evaluate differences between the two groups. Univariate and multivariate Cox regression analyses were performed to identify prognostic factors for survival outcomes and graphically plotted using forest plots. Only variables in the univariate analysis at *p* < 0.05 were included in the multivariate analysis. To further evaluate the prognostic significance of the L/S ratio, Cox regression models with and without the L/S ratio were constructed to predict 10‐year survival outcomes. The performance of these models was estimated via the concordance index (C‐index) and the time‐dependent area under the curve (tAUC) of tROC which could estimate t‐year survival predicting performance for right‐censored data. The DeLong test was performed to assess the differences between the tAUCs of different models. In addition, to visualize the continuous performances of prediction models between the two groups from 5–15 years, the integrated tAUCs (iAUCs) were calculated. Considering the limited sample size, we performed leave‐one‐out cross‐validation (LOOCV) to validate the performance of these models. All statistical calculations were performed using R version 3.6.3 (R Foundation for Statistical Computing) using the survival, survminer, rms, survivalROC, and timeROC and survAUC packages.

## RESULTS

### Threshold of L/S ratio

An L/S ratio of 1.39 showed the best discriminative performance for 10‐year OS by tROC curve (tAUC = 0.734) (Figure E2). Therefore, an L/S ratio of 1.39 was used as a threshold for clinical significance in subsequent analyses. The groups of higher (*n* = 42) and lower (*n* = 94) L/S ratios were defined as an L/S ratio >1.39 and an L/S ratio ≤ 1.39, respectively. Furthermore, by using the same statistical methods, patients with R0 resection thymoma (*n* = 117) were divided into higher and lower L/S ratio groups (*n* = 21 vs. 96) according to the threshold value of 1.54 (tAUC = 0.697) (Figure E2).

### Patient clinicopathological characteristics

Patient clinicopathological characteristics between different L/S ratio groups are shown in Table 1. In total, 62 male (45.6%) and 74 female (54.4%) patients with a median age of 60 years (range, 24 to 83 years) were included. There were 22 (16.2%) patients who underwent postoperative adjuvant therapy. Of these patients, 12 patients were conducted radiotherapy with the dose from 40 Gy to 70 Gy, and 10 patients were administered 1–4 cycles of chemotherapy with the ADOC regimen including cisplatin (50 mg/m^2^), doxorubicin (40 mg/m^2^), vincristine (0.6 mg/m^2^), and cyclophosphamide (700 mg/m^2^), or TP regimen of cisplatin (50 mg/m^2^) and paclitaxel (225 mg/m^2^). In our study, only 10 (7.4%) patients were diagnosed with thymic carcinoma, including seven squamous cell carcinomas, two undifferentiated carcinomas, and one adenocarcinoma. Although subtle differences existed between the two groups, the clinicopathological features were generally comparable between the two groups. Patients with a higher L/S ratio had a greater incidence of disease progression than patients with a lower L/S ratio (*p* = 0.001). After excluding patients with either thymic carcinoma or non‐R0, a significant difference between patients with higher and lower L/S ratios was still observed only for recurrence (*p* = 0.003) (Table E1). For these thymoma patients with recurrence (*n* = 14), seven patients presented pleural dissemination, in which five patients underwent pleuropneumonectomy or pleurectomy, and the other two patients were conducted radiotherapy (45Gy).

**TABLE 1:** Clinicopathological characteristics of thymic epithelial tumors

Characteristics	All patients (*N* = 136)	Higher L/S ratio^e^ (*N* = 42)	Lower L/S ratio^f^ (*N* = 94)	*p‐*value
Age (years) (median) (rang)	60 (24–83)	60 (24–81)	60 (29–83)	0.721^a^
Sex				0.122^b^
Male	62 (45.6%)	15 (35.7%)	47 (50.0%)	
Female	74 (54.4%)	27 (64.3%)	47 (50.0%)	
Surgical approach				0.323^d^
Median sternotomy	123 (90.4%)	38 (90.5%)	85 (90.4%)	
VATS‐transthoracic	9 (6.6%)	4 (9.5%)	5 (5.3%)	
VATS‐subxiphoid	4 (2.9%)	0 (0)	4 (4.3%)	
Adjuvant therapy				0.266^b^
Yes	22 (16.2%)	9 (21.4%)	13 (13.8%)	
No	114 (83.8%)	33 (78.6%)	81 (86.2%)	
MG				0.627^b^
Yes	26 (19.1%)	7 (16.7%)	19 (20.2%)	
No	110 (80.9%)	35 (83.3%)	75 (79.8%)	
WHO type^g^				0.315^c^
thymoma (A/AB/B)	126 (92.6%)	37 (88.1%)	89 (94.7%)	
Thymic carcinoma	10 (7.4%)	5 (11.9%)	5 (5.3%)	
TNM stage ^△^				0.08^d^
I	106 (77.9%)	30 (71.4%)	76 (80.9%)	
II	3 (2.2%)	1 (2.4%)	2 (2.1%)	
III	14 (10.3%)	3 (7.1%)	11 (11.7%)	
IV	13 (9.6%)	8 (19.0%)	5 (5.3%)	
Surgical radicality				0.894^c^
R0	124 (91.2%)	38 (90.5%)	86 (91.5%)	
non‐R0	12 (8.8%)	4 (9.5%)	8 (8.5%)	
Disease progression				0.001^b,^, *
Yes	23 (16.9%)	14 (33.3%)	9 (9.6%)	
No	113 (83.1%)	28 (66.7%)	85 (90.4%)	

Abbreviations: L/S ratio, long‐to‐short axis ratio; MG, myasthenia gravis; non‐R0, incomplete resection on both microscopy and macroscopy; R0, microscopically complete resection; TC, thymic carcinoma; TNM, tumor, node, metastasis; VATS, video‐assisted thoracoscopic surgery; WHO, World Health Organization.

^a^ Independent‐sample *t* test.

^b^ Pearson's chi‐square test.

^c^ Continuity adjusted chi‐square test.

^d^ Fisher's exact test.

^e^ L/S ratio >1.39.

^f^ L/S ratio ≤1.39.

^g^ WHO type (fifth edition, 2021), ^△^ TNM staging (AJCC/UICC, eighth^t^ edition).

* *p* < 0.05.

### Survival outcomes

**FIGURE 1:**
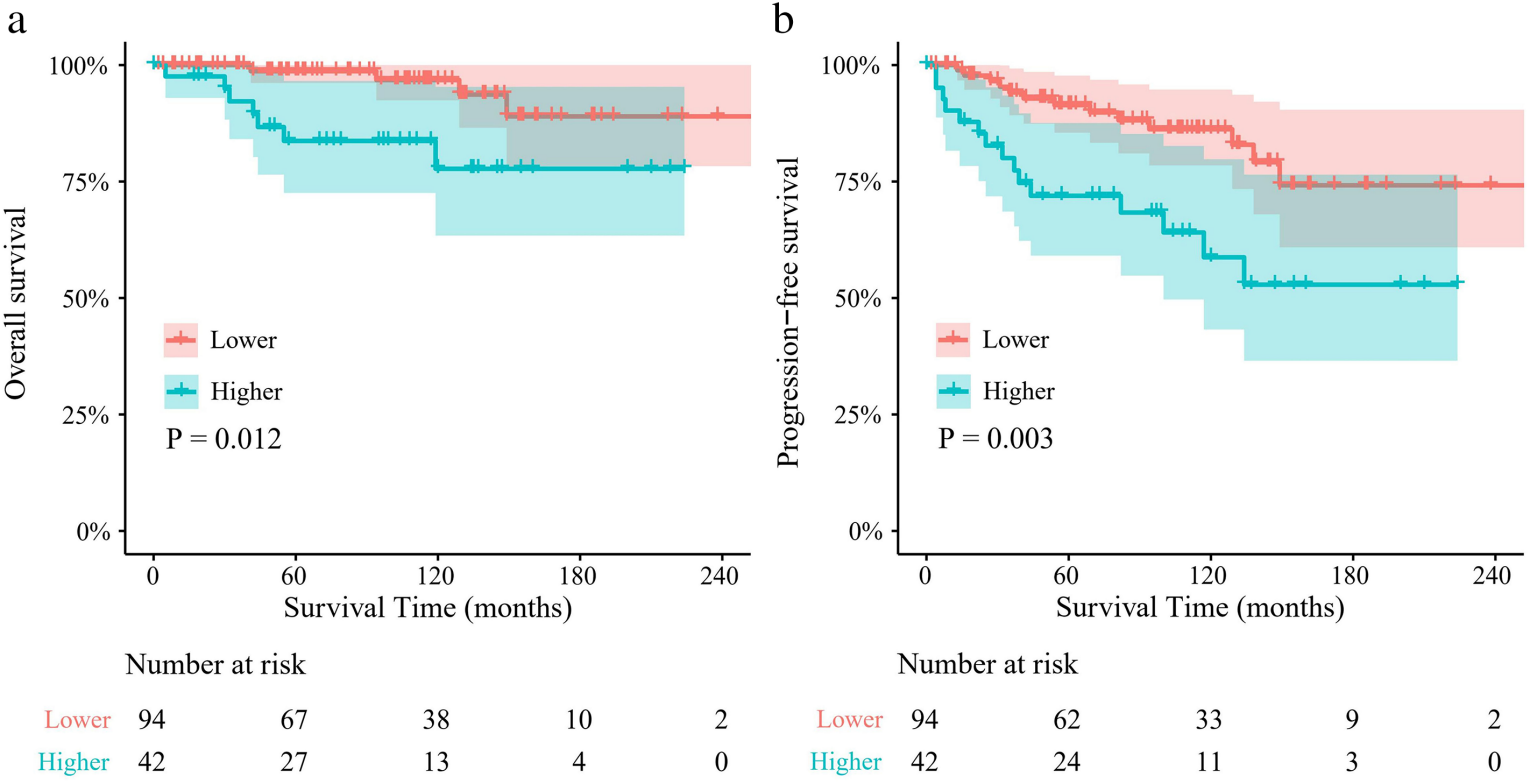
The Kaplan‐Meier curves for the survival outcomes of patients with thymic epithelial tumors in different groups. (a) Patients with higher tumor long‐to‐short axis (L/S) ratio had worse overall survival (OS) and (b) progression‐free survival (PFS) than patients with lower L/S ratio (*p* = 0.012 and *p* = 0.003, respectively). The mean survival times of OS and PFS between the two groups (higher vs. lower L/S ratio) were 187.0 ± 12.6 months versus 244.7 ± 7.5 months and 145.9 ± 15.5 months versus 216.7 ± 11.1 months, respectively

With a mean follow‐up duration of 108 months (range: 0–260 months), the 10‐year OS and PFS rates of patients with higher and lower L/S ratios were 78.5% versus 96.7% and 62.3% versus 89.2%, respectively (Table E2). The survival curves stratified by the threshold L/S ratio of 1.39 are shown in Figure 1. Patients with an L/S ratio of >1.39 showed significantly worse mean OS (187.0 ± 12.6 months vs. 244.7 ± 7.5 months) and PFS (145.9 ± 15.5 months vs. 216.7 ± 11.1 months) than patients with an L/S ratio of ≤ 1.39 (*p* = 0.012 and 0.003, respectively). Additionally, for R0 resected thymoma patients, a significant difference of prognosis was observed between the two groups, whereby a higher L/S ratio was associated with poorer OS and DFS (*p* < 0.05) (Figure E3).

### Prognostic factors for survival outcomes

**FIGURE 2:**
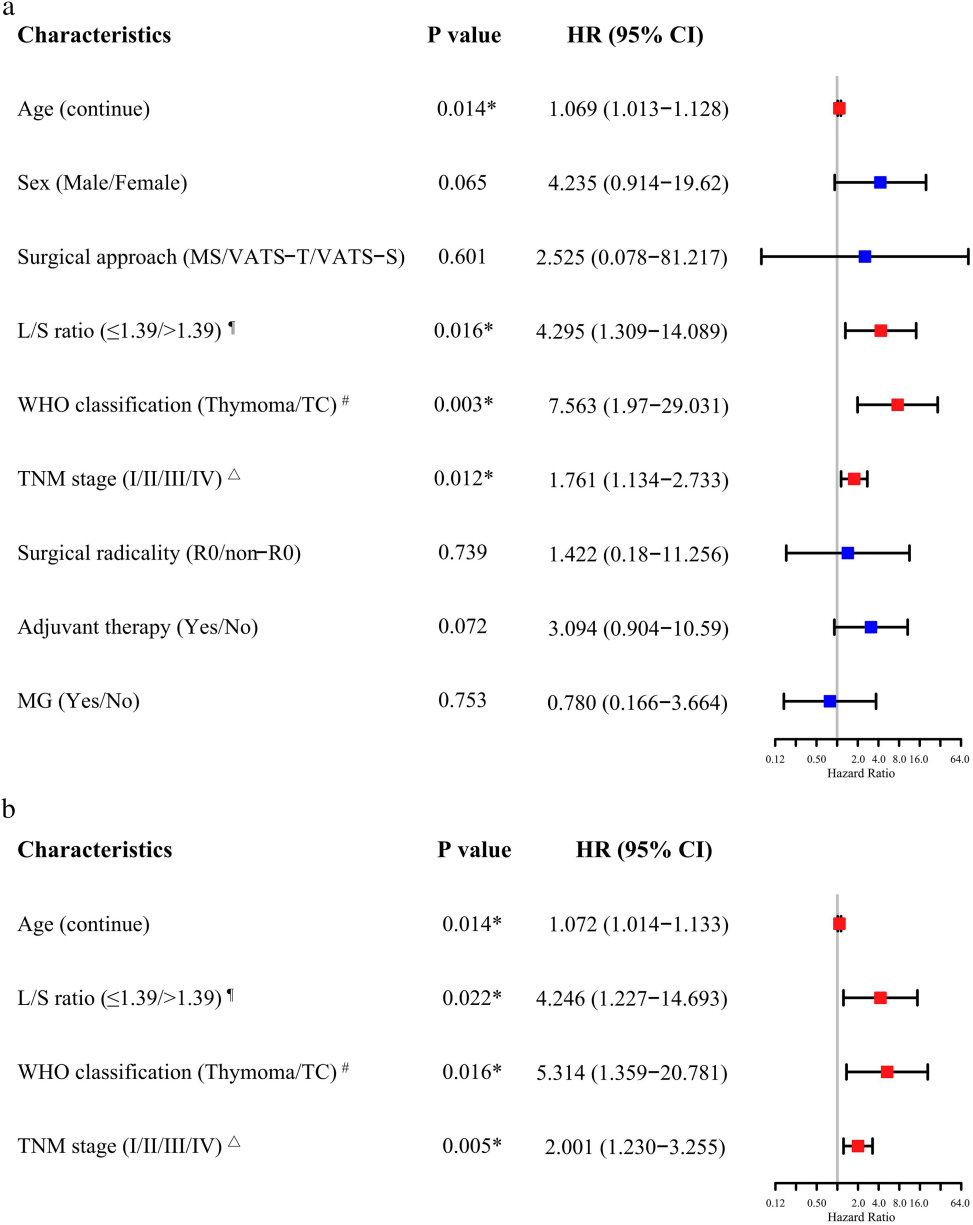
The univariate and multivariate analysis with forest plots of prognostic factors according to overall survival in thymic epithelial tumors. * *p* < 0.05, ^¶^the optimal threshold = 1.39, ^#^WHO type (fifth edition, 2021), ^△^TNM staging (AJCC/UICC, eighth edition). L/S ratio, long‐to‐short axis; WHO, World Health Organization; TC, thymic carcinoma; TNM, tumor, node, metastasis; R0, microscopically complete; non‐R0, incomplete on both microscopy and macroscopy; MG, myasthenia gravis; MS, median sternotomy; VATS‐T, video‐assisted thoracoscopic surgery‐transthoracic; VATS‐S, video‐assisted thoracoscopic surgery‐subxiphoid; HR, hazard ratio; CI, confidence interval

The results of the univariate and multivariate analysis for OS are shown in Figure 2. Age, L/S ratio, WHO classification and TNM stage were associated with OS in the univariate analysis (*p* < 0.05) (Figure 2a). In the multivariate analysis, increased age (hazard ratio [HR] 1.065, 95% confidence interval [CI]: 1.008–1.126; *p* = 0.026), L/S ratio > 1.39 (HR 4.693, 95% CI: 1.331–16.544; *p* = 0.016), thymic carcinoma (HR 4.773, 95% CI: 1.237–18.421; *p* = 0.023) and more advanced TNM stage (HR 4.411, 95% CI: 1.281–15.183; *p* = 0.019) emerged as independent adverse prognostic factors (Figure 2b).

**FIGURE 3:**
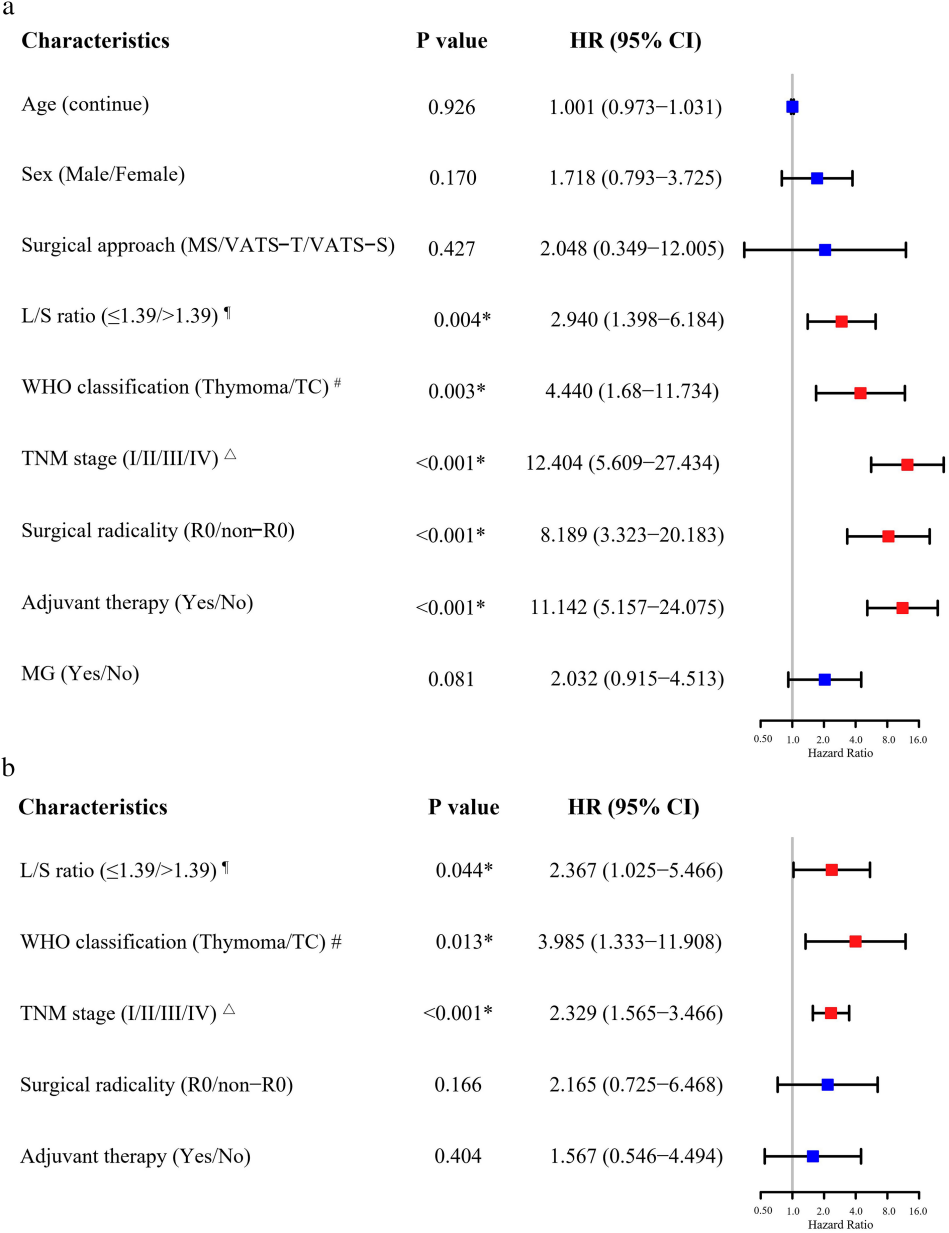
The univariate and multivariate analysis in forest plots of prognostic factors according to progression‐free survival in thymic epithelial tumors. * *p* < 0.05, ^¶^ The optimal threshold = 1.39. ^#^WHO type (fifth edition, 2021), ^△^TNM staging (AJCC/UICC, eighth edition). L/S, long‐to‐short axis; WHO, World Health Organization; TC, thymic carcinoma; TNM, tumor, node, metastasis; R0, microscopically complete resection; non‐R0, incomplete resection on both microscopy and macroscopy; MS, median sternotomy; VATS‐T, video‐assisted thoracoscopic surgery‐transthoracic; VATS‐S, video‐assisted thoracoscopic surgery‐subxiphoid; MG, myasthenia gravis; HR, hazard ratio; CI, confidence interval

**FIGURE 4:**
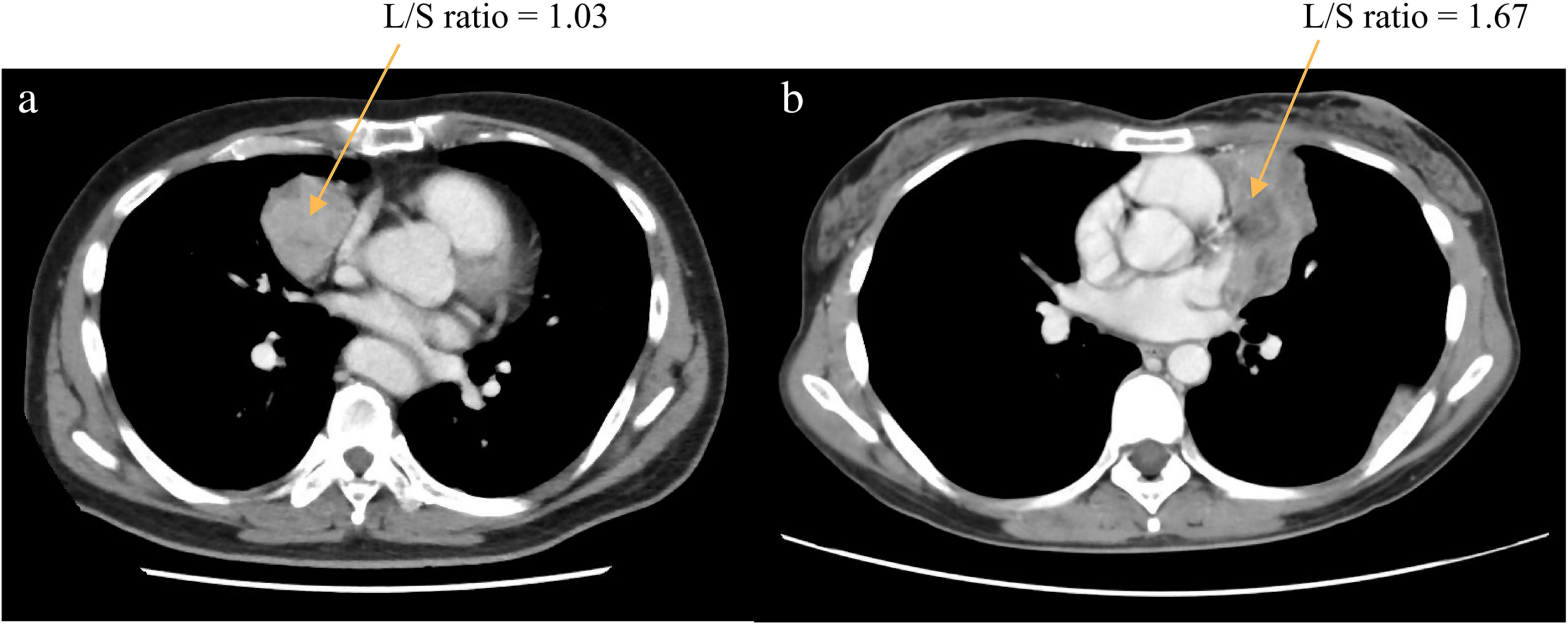
Computed tomography images of two representative patients for L/S ratio. An example is a 61‐year‐old man with a low L/S ratio (1.03) thymoma (maximum diameter = 8 cm; minimum diameter = 7.8 cm) and TNM stage I who was still alive at the last follow‐up (a). Another example is a 34‐year‐old woman with a high L/S ratio (1.67) thymoma (maximum diameter = 12.5 cm; minimum diameter = 7.5 cm) and TNM stage II who was dead at the last follow‐up (b). L/S, long‐to‐short axis

According to the univariable analysis of PFS, the L/S ratio, WHO classification, TNM stage, surgical radicality, and adjuvant therapy were significant prognostic factors of PFS (*p* < 0.05) (Figure 3a). After adjusting for significant clinical variables, the multivariable analysis revealed that the L/S ratio (HR 2.892, 95% CI: 1.257–6.651; *p* = 0.012), WHO classification (HR 3.164, 95% CI: 1.102–9.086; *p* = 0.032), and TNM stage (HR 7.841, 95% CI: 2.807–21.905; *p* < 0.001) were independent prognostic factors for PFS (Figure 3b). Clinical examples of L/S ratio are shown in Figure 4.

In patients with thymoma who underwent R0 resection, the multivariate analysis demonstrated that the age, L/S ratio, and TNM stage were independent prognostic factors for both OS and DFS (*p* < 0.05) (Table E3).

### Significance of the L/S ratio in prediction models

**FIGURE 5:**
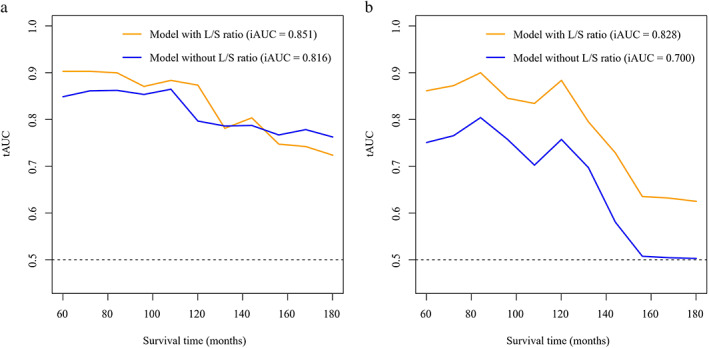
The continuous performances of models to predict survival outcomes. The iAUC of models with an L/S ratio were higher than those of models without an L/S ratio for predicting (a) overall survival (0.846 vs. 0.789) and (b) progression‐free survival (0.786 vs. 0.695) at 5–15 years. iAUC, integrated time‐dependent area under the receiver operator characteristic curve; L/S, long‐to‐short axis

We further estimated the significance of the Cox regression models with and without the L/S ratio for survival outcomes **(**Table 2 and Figure E4). The C‐index for OS and PFS in these models with and without L/S ratios was 0.897 versus 0.856 and 0.873 versus 0.829, respectively. Moreover, the tAUCs of 10‐year OS and PFS showed a higher value in models with L/S ratio after LOOCV than models without L/S ratio (0.844 vs. 0.738 and 0.844 vs. 0.715, respectively). The Delong test showed a higher tAUC value in the model with L/S ratio, even though statistical significance between the two groups was not observed (*p* = 0.260). Furthermore, the performance of models with the L/S ratio was significantly better than that without the ratio (*p* = 0.037). Additionally, the iAUC of models with an L/S ratio were higher than those of models without an L/S ratio for predicting OS (0.846 vs. 0.789) and PFS (0.786 vs. 0.695) at 5–15 years (Figure 5).

**TABLE 2:** Performances in models with or without long‐to‐short axis ratio to predict survival outcomes

Outcomes	Modeling parameters	C‐index (95% CI)	tAUC (95% CI)	*p‐*value^c^
10‐year OS	Age, WHO type, TNM stage, and **L/S ratio**	0.897 (0.82–0.972)	0.873 (0.765–0.982)	0.194^a^
	Age, WHO type, and TNM stage	0.856 (0.744–0.968)	0.798 (0.610–0.985)	
10‐year PFS	WHO type, TNM stage, and **L/S ratio**	0.873 (0.820–0.926)	0.884 (0.770–0.997)	0.040^b^ ^,^ *
	WHO type and TNM stage	0.829 (0.754–0.905)	0.757 (0.577–0.937)	

*Note*: All results were validated by leave‐one‐out cross‐validation.

Abbreviations: CI, confidence interval; C‐index, concordance index; L/S ratio, long‐to‐short axis ratio; OS, overall survival; PFS, progression‐free survival; tAUC, time‐dependent area under the receiver operator curve; TNM, tumor, node, metastasis; WHO, World Health Organization.

^a^ The difference of tAUC between models with and without L/S ratio.

^b^ The difference of tAUC between models with and without L/S ratio.

^c^ Using DeLong test to assess the differences between the tAUCs of different models.

* *p* < 0.05.

## DISCUSSION

Considering the heterogeneous shape of TETs, such as round, oval, and irregular/lobulated, the current study first focused on the pathological tumor L/S ratio for patients with TETs and revealed the following findings: (i) An L/S ratio >1.39 was significantly correlated with a greater incidence of disease progression and poorer survival outcomes. (ii) In addition to the TNM stage and WHO classification, the L/S ratio was the only independent prognostic factor for OS and PFS. (iii) The prediction models with the L/S ratio achieved better performance than those without the L/S ratio for 5‐ to 15‐year survival outcomes.

In many previous studies, factors representing tumor size, such as maximum diameter, was regarded as a significant prognostic factor for patients with TETs.^21^ However, the importance of tumor shape was usually ignored, compared with tumor maximum diameter. After analyzing the relationship between maximum diameter and L/S ratio, we found that Pearson's correlation coefficient of the two factors was only 0.177 (Figure E5). The low correlation illustrated that tumor shape and tumor size represent different prognostic information. In addition, we further found that the HR of L/S ratio was higher than that of tumor maximum diameter (4.246 vs. 1.246, Table E4). Therefore, the L/S ratio may provide more prognostic information than tumor maximum diameter for patients with TETs. Previous studies regarding TETs defined the tumor shape by a rough L/S ratio: L/S ratio <1.5 for round tumors, 1.5‐3.0 for oval tumor, and >3.0 for irregular/lobulated tumor.^14^, ^22^, ^23^, ^24^ Qu et al.^14^ demonstrated that tumors were in an oval or round shape (L/S ratio < 3.0) in early stages (I/II) but were usually irregularly shaped or lobulated in advanced stages (III/IV) from computed tomography (CT) images. By analyzing pathological data in our current study, no correlation was found between the L/S ratio (≤1.39/>1.39) and TNM stage (I, II, III, and IV). The difference may be attributed to the fact that all the previous studies used CT scans to define the L/S ratio. However, the tumor size of TETs on CT scans was 0.4 cm smaller than that of pathology assessments.^18^ In our case series, the mean tumor size (maximum length) of TETs on CT scan showed approximately 1.0 cm shorter than that measured by pathology (4.6 ± 2.3cm vs. 5.6 ± 2.9 cm) **(**Table E5). In addition, the L/S ratio from CT scans often measures cross‐sectional but not sagittal data, which may not represent an accurate ratio of the longest and shortest diameters of tumors.

Furthermore, our results were in accordance with findings from Tomiyama et al.^23^ and Jeong et al.^25^ that the L/S ratio was comparable in the WHO classification. Nevertheless, Yanagawa and colleagues^26^ indicated that there were greater numbers of thymomas with a round shape than thymic carcinomas. There were also inconsistencies in surgical radicality. Shen et al.^12^ assessed the tumor shape between patients with R0 and non‐R0 resection and concluded that the round tumors tend to be more often associated with R0 resection, but no difference was found in our study. The inconsistent results may be related to the extended thymectomy performed in all of our cases. In contrast, palliative surgery, biopsy only, debulking of the tumor were performed in Shen and colleagues' series. In the current study, the oncological behavioral characteristics were not significantly correlated with L/S ratio, but the patients with higher L/S ratio showed more advanced TNM stage than those with lower L/S ratio.

Regarding recurrence, Jeong et al.^25^ found a greater frequency of recurrence or metastasis was associated with oval‐shaped TETs. However, Priola and colleagues revealed no correlation between tumor shape and relapse in patients with thymoma.^24^ In our study, patients who exhibited disease progression were more often in the high L/S group, which was in accordance with findings of Jeong et al.^25^ Even among patients with R0 resection thymomas in our study, a higher L/S ratio was associated with a greater incidence of recurrence than a lower L/S ratio.

Due to highly favorable outcomes associated with TETs, previous studies on TETs only elucidated the relationship of tumor shape with Masaoka‐Koga/TNM staging, WHO classification, or tumor invasion but not survival outcomes.^12^, ^23^, ^24^, ^25^, ^26^ However, the literature on lung adenocarcinoma has demonstrated the significance of tumor shape described by the L/S ratio with respect to survival outcomes and found the 5‐year OS and DFS rates in patients with round tumors were worse than those in patients with ellipse tumors.^11^ In contrast with lung adenocarcinoma, our findings showed that TET patients with a higher L/S ratio had worse survival outcomes than those with a lower L/S ratio. The possible biological rationale may be that the high L/S ratio TETs (high‐risk tumors) often show less or no fibrous septum, which may restrict the tumor focus on growing in one direction. On the contrary, most of the low L/S ratio TETs (low‐risk tumor) present more fibrous septum, and the tumor may extend in one direction where no septum can limit their invasion (Figure E6).

Numerous studies have demonstrated the importance of clinicopathological features as predictive factors for survival outcomes, including age,^27^, ^28^ tumor length/diameter,^5^, ^18^ tumor stage,^10^, ^29^ and WHO type.^7^, ^29^ Therefore, novel parameters have been explored to achieve a clear understanding of the prognostic factors of TETs and improve survival outcomes.^8^, ^9^, ^30^ In this present study, we first reported that the pathological tumor L/S ratio was a prognostic factor for survival outcomes in TETs. Furthermore, even when we included only thymoma patients who underwent R0 resection, we found a higher L/S ratio can predict a worse prognosis. In addition, we evaluated the prediction performance with and without the L/S ratio in a Cox regression model. Not surprisingly, the performance of prediction models with the L/S ratio was better than that without for survival outcomes during the follow‐up duration from 5 to 15 years, which further revealed that the L/S ratio was a crucial predicting marker in TETs.

Our recent studies have concurrently verified the significance of the TNM stage in predicting the prognosis of patients with TETs.^8^, ^9^ In the current study, we restaged the TNM stage and found that the TNM stage was another independent prognostic factor for all survival outcomes in TETs other than the L/S ratio. Thus, our results supported previous studies showing that the TNM stage was one of the independent prognostic factors for survival outcomes in TETs.^8^, ^9^, ^31^, ^32^

The WHO classification of TETs divided into thymoma and thymic carcinoma has been proven to be a valuable predicting marker for survival outcomes.^7^, ^21^, ^33^ Our results were in line with the previous findings and demonstrated the WHO classification of TETs as a prognostic factor for OS and PFS. In this cohort, age was only shown as one of the independent prognostic factors for OS in patients with TETs but an independent prognostic factor for both OS and DFS in patients with R0 resection thymoma. Understandably, patients with TETs often presented a long survival even after recurrence.^28^ A previous study demonstrated that thymoma was responsible for only 23.1%–37.9% of all deaths during the follow‐up period.^34^, ^35^ During the long survival duration, many patients, especially patients with thymoma, died of causes other than tumors, which leads to age being an independent prognostic factor.

The current study has several limitations that should be mentioned. First, this was a retrospective case series from a single center with a small sample size which spanned more than 20 years. Although we used rigorous selection procedures and multivariate analysis to adjust for the covariates, inherent selection and verification biases still existed due to the unbalanced numbers of patients in the groups. Due to the excellent survivability and rarity of TETs, a randomized controlled trial may be more challenging to execute. We believe that the results of our pilot study will encourage future research; a multicenter analysis using larger cohorts is essential to confirm our findings. Second, due to there being only six patients who died of recurrent disease during the follow‐up period (not shown in our results), we did not describe the surgical intervention for recurrent patients who may have experienced better survival through surgery than conservative treatment. Third, the threshold value of the L/S ratio could not be firmly established from this pilot study. Because the current threshold value of the L/S ratio was a novel predictive marker of TETs, a more precise threshold should be determined using a larger population. Despite these limitations, this study has a certain value as it is the first study to demonstrate the possibility of predicting prognosis using the L/S ratio.

In conclusion, our study is the first to demonstrate that pathological tumor L/S ratio > 1.39 tended to be associated with a higher incidence of disease progression and worse survival outcomes of OS and PFS in patients with TETs. The L/S ratio was an independent prognostic factor for the survival outcomes in TET patients. This parameter may help clinicians develop optimal postoperative strategies and follow‐up surveillance protocols for individual patients.

## CONFLICT OF INTEREST

The authors declare no conflict of interest.

## Supporting information


**Appendix S1** Supporting Information
