# Supplementary material for: Pathological tumor long‐to‐short axis ratio as a prognostic factor in patients with thymic epithelial tumors
Source: Thorac Cancer. 2022 Jul 21;13(17):2489–98. doi: 10.1111/1759-7714.14582 (PMC9436687; doi:10.1111/1759-7714.14582)
Supplement: Supplementary file 1 — Appendix S1 Supporting Information [file TCA-13-2489-s001.docx]

**Supplementary material**

**TABLE E1: Clinicopathological characteristics in different** **groups of** **R0 resection thymoma**

| **Characteristics** | **All patients**  **(N = 117)** | **Higher L/S ratio ^†^**  **(N = 21)** | **Lower L/S ratio ^‡^**  **(N = 96)** | ***P* value** |
| --- | --- | --- | --- | --- |
| Age (y) (median) (rang) | 59 (24-83) | 52 (24-78) | 60.5 (29-83) | 0.175 ^a^ |
| Sex |  |  |  | 0.366 ^b^ |
| Male | 55 (47.0%) | 8 (38.1%) | 47 (49.0%) |  |
| Female | 62 (53.0%) | 13 (61.9%) | 49 (51.0%) |  |
| Surgical approach |  |  |  | 0.853 ^d^ |
| Median sternotomy | 104 (88.9%) | 19 (90.5%) | 85 (88.5%) |  |
| VATS-Transthoracic | 9 (7.7%) | 2 (9.5%) | 7 (7.3%) |  |
| VATS-Subxiphoid | 4 (3.4%) | 0 (0) | 4 (4.2%) |  |
| Adjuvant therapy |  |  |  | 0.543 ^c^ |
| Yes | 10 (8.5%) | 3 (14.3%) | 7 (7.3%) |  |
| No | 107 (91.5%) | 18 (85.7%) | 89 (92.7%) |  |
| MG |  |  |  | 0.761 ^c^ |
| Yes | 22 (18.8%) | 3 (14.3%) | 19 (19.8%) |  |
| No | 95 (81.2%) | 18 (85.7%) | 77 (80.2%) |  |
| TNM stage ^△^ |  |  |  | 0.134 ^d^ |
| Ⅰ- | 101 (86.3%) | 18 (85.7%) | 83 (86.5%) |  |
| Ⅱ | 3 (2.65) | 0 (0) | 3 (3.1%) |  |
| Ⅲ- | 7 (6.0%) | 0 (0) | 7 (7.3%) |  |
| Ⅳ | 6 (5.1%) | 3 (14.3%) | 3 (3.1%) |  |
| Recurrence |  |  |  | 0.003 ^c,*^ |
| Yes | 14 (12.0%) | 7 (33.3%) | 7 (7.3%) |  |
| No | 103 (88.0%) | 14 (66.7%) | 89 (92.7%) |  |

^*^ *P* < 0.05, ^†^ L/S ratio > 1.54, ^‡^ L/S ratio ≤ 1.54,

^△^ TNM staging (AJCC/UICC, 8^th^ edition),

^a^: Independent-sample t test, ^b^: Pearson’s chi-square test, ^c^: Continuity adjusted chi-square, ^d^ Fisher’s exact test.

R0, microscopically complete resection; MG, myasthenia gravis; VATS, video-assisted thoracoscopic surgery, TNM, tumor, node, metastasis.

**TABLE E2. The 10-year survival rates in different groups**

| **Survival** | **Higher L/S ratio** | |  | **Lower L/S ratio** | |
| --- | --- | --- | --- | --- | --- |
|  | **Survival rate** | **95% CI** |  | **Survival rate** | **95% CI** |
| 10-year OS | 0.785 | 0.583-0.897 |  | 0.967 | 0.873-0.992 |
| 10-year PFS | 0.623 | 0.424-0.771 |  | 0.892 | 0.794-0.945 |

OS, overall survival; PFS, progression-free survival; DFS, disease-free survival; L/S ratio, long-to-short axis ratio; CI, confidence interval.

**TABLE E3. Multivariate analysis of prognostic factors for overall survival and disease-free survival in patients with R0 resection thymoma.**

| **Characteristics** | **HR** | **95% CI** | **P value** |
| --- | --- | --- | --- |
| **OS** |  |  |  |
| Age (continue) | 1.179 | 1.066, 1.304 | **0.001 ^*^** |
| Sex (Male/Female) | 2.724 | 0.384, 19.303 | 0.316 |
| Surgical approach (MS/VATS-T/VATS-S) | 47.206 | 0.013, 170395.081 | 0.356 |
| L/S (≤1.54/>1.54) ^¶^ | 20.375 | 2.688, 154.419 | **0.004 ^*^** |
| TNM stage (Ⅰ/Ⅱ/Ⅲ/Ⅳ) ^△^ | 4.000 | 1.127, 14.197 | **0.032 ^*^** |
| Adjuvant therapy (Yes/No) | 0.469 | 0.012, 17.986 | 0.684 |
| MG (Yes/No) | 2.348 | 0.360, 15.290 | 0.372 |
| **DFS** |  |  |  |
| Age (continue) | 1.059 | 1.015, 1.105 | **0.008 ^*^** |
| Sex (Male/Female) | 0.909 | 0.351, 2.357 | 0.845 |
| Surgical approach (MS/VATS-T/VATS-S) | 6.925 | 0.253, 189.460 | 0.252 |
| L/S (≤1.54/>1.54) ^¶^ | 3.997 | 1.406, 11.366 | **0.009 ^*^** |
| TNM stage (Ⅰ/Ⅱ/Ⅲ/Ⅳ) ^△^ | 2.767 | 1.451, 5.277 | **0.002 ^*^** |
| Adjuvant therapy (Yes/No) | 1.495 | 0.216, 10.348 | 0.683 |
| MG (Yes/No) | 4.095 | 1.372, 12.218 | **0.011 ^*^** |

^*^ *P* < 0.05, ^¶^ The optimal threshold = 1.54, ^△^ TNM staging (AJCC/UICC, 8^th^ edition).

OS, overall survival DFS; and disease-free survival; HR, hazard ratio; CI, confidence interval; MS, median sternotomy; VATS-T, video-assisted thoracoscopic surgery - Transthoracic; VATS-S, video-assisted thoracoscopic surgery - Subxiphoid; L/S ratio; long-to-short axis ratio; MG, myasthenia gravis.

**TABLE E4. The multivariate analysis of prognostic factors including tumor maximum diameter to overall survival in thymic epithelial tumors.**

| **Characteristics** | **HR (95% CI)** | **P value** |
| --- | --- | --- |
| Age (continue) | 1.084 (1.019-1.154) | 0.011 |
| Tumor maximum diameter | 1.246 (1.027-1.513) | 0.026 |
| WHO classification (Thymoma/TC) ^#^ | 7.888 (1.807-34.437) | 0.006 |
| TNM stage (Ⅰ/Ⅱ/Ⅲ/Ⅳ) ^△^ | 1.852 (0.469-7.313) | 0.379 |

^*^ *P* < 0.05, ^#^ WHO type (5th edition, 2021), ^△^ TNM staging (AJCC/UICC, 8th edition).

L/S ratio, long-to-short axis; WHO, World Health Organization; TNM, tumor, node, metastasis; HR, hazard ratio; CI, confidence interval.

**TABLE E5. The tumor size measured by computed tomography and pathology**

| **Tumor size** | **Computed tomography** | **Pathology** |
| --- | --- | --- |
| Mean ± Standard Deviation (cm) | 4.6 ± 2.3 | 5.6 ± 2.9 |
| Median (Range) (cm) | 4.2 (1.0, 17.0) | 5.0 (1.2, 20.0) |

**Figure Legends**

**
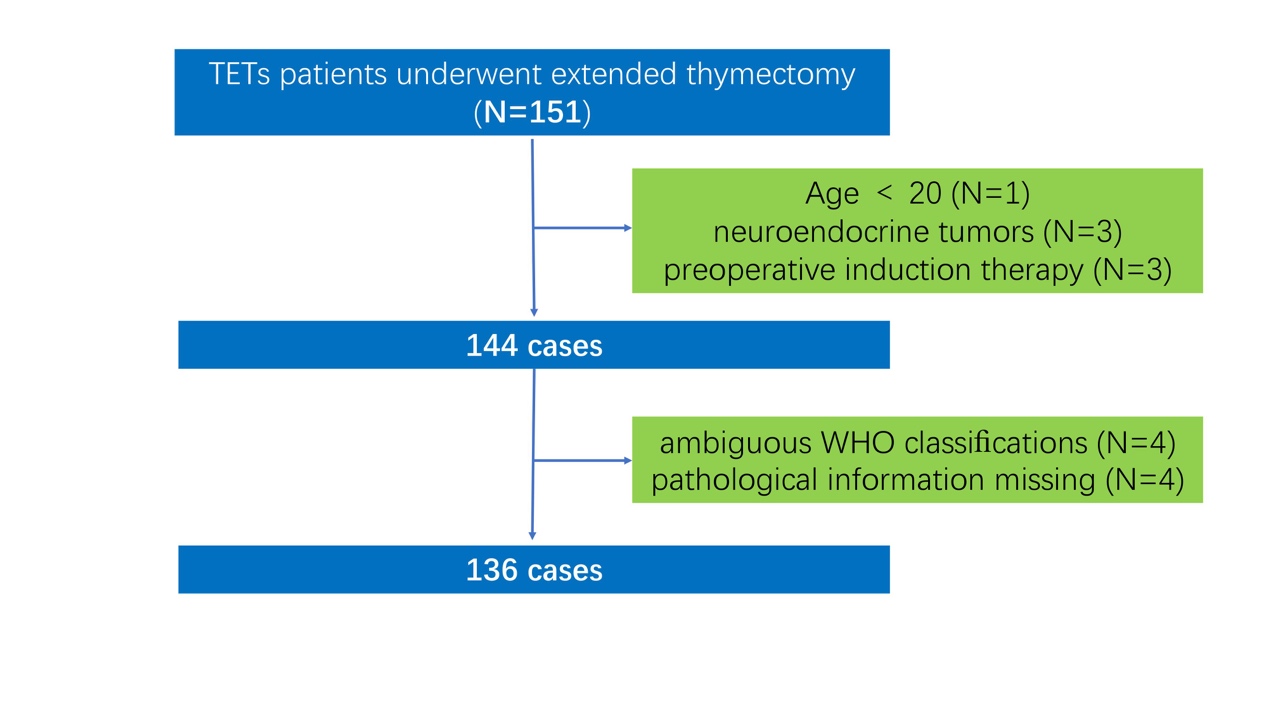
**

**FIGURE E1 Flow chart of patient recruitment and exclusion**

Between January 1999 and December 2019, 151 patients were enrolled. Of these, one with age less than 20, three with neuroendocrine tumors, three who received preoperative induction therapy, four with ambiguous WHO classiﬁcations, and four who did not have detailed information regarding the pathological tumor size were excluded from the study, 136 patients were ultimately enrolled, and their clinicopathological features and survival outcomes were evaluated retrospectively.

TETs, thymic epithelial tumors; WHO, World Health Organization.


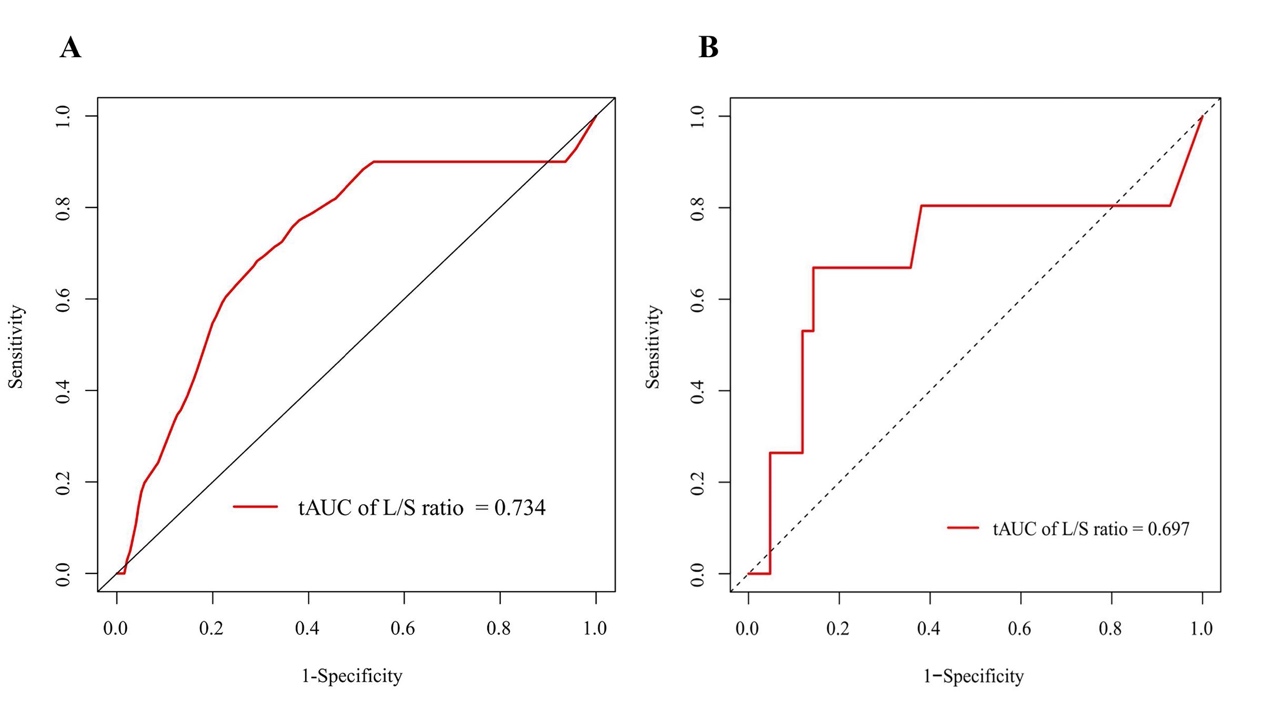


**FIGURE E2. Time-dependent receiver operator characteristic curve**

**(A)** The optimal threshold of the L/S ratio from the tROC curve for 10-year overall survival in thymic epithelial tumors was at 1.39. The tAUC of L/S ratio was 0.734 (95% CI, 0.508-0.960) in thymic epithelial tumors.

**(B)** The optimal threshold of L/S ratio from the tROC curve for 10-year overall survival in R0 resection thymoma was at 1.54. The tAUC of L/S ratio was 0.697 (95% CI, 0.398-0.996).

L/S, long-to-short axis; tROC, time-dependent receiver operator characteristic; tAUC, time-dependent area under the curve of tROC.


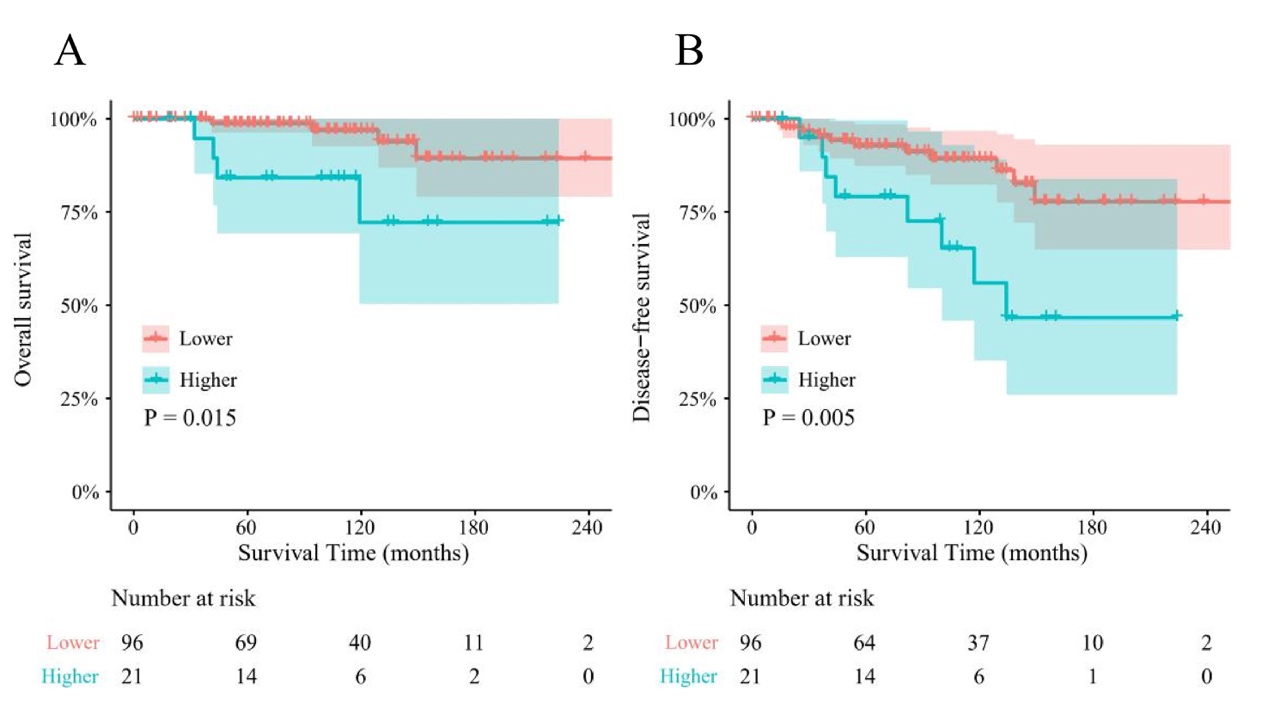


**FIGURE E3**. **Kaplan-Meier curves for the survival outcomes** **of patients with R0 resection thymoma in different groups**

(**A**) Patients with a higher long-to-short axis ratio had worse overall survival (OS) and (**B**) disease-free survival (DFS) than patients with lower long-to-short axis ratio (*P* = 0.015 and P = 0.005). The mean survival times of OS and DFS between the two groups (higher- vs. lower- L/S ratio) were 182.2 ± 18.4 months vs. 245.2 ± 7.3 months and 148.2 ± 19.5 months vs. 223.7 ± 10.3 months, respectively.

**
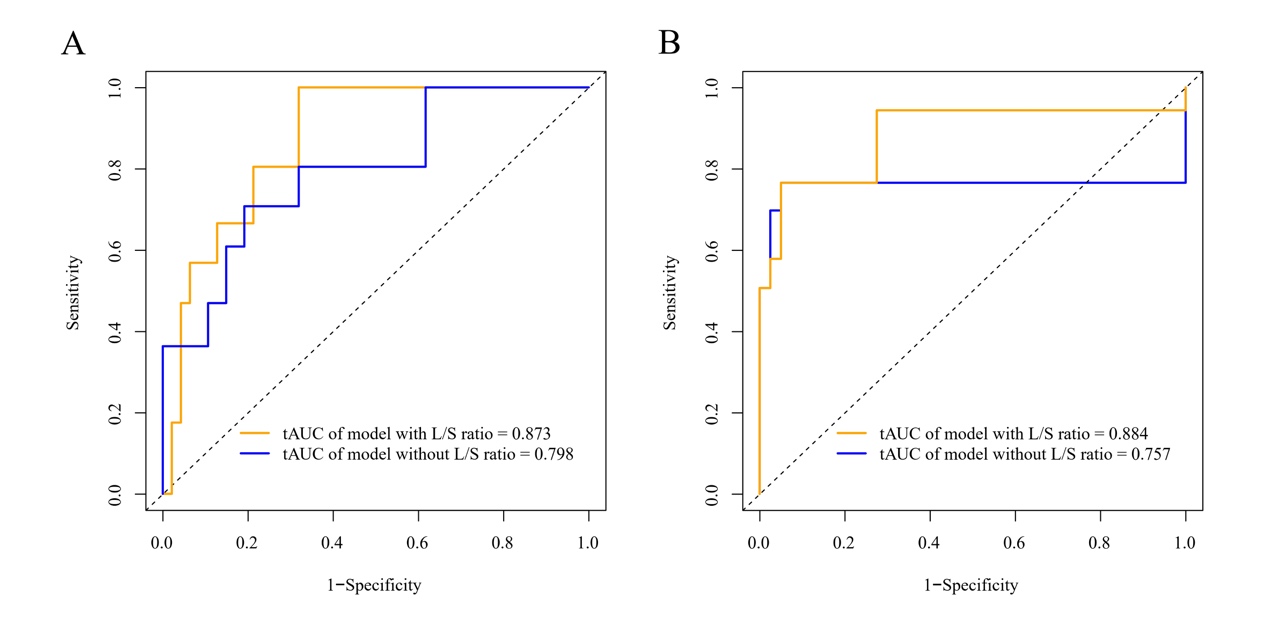
**

**FIGURE E4. Time-dependent receiver operator characteristic curves of models with or without L/S ratio to predict 10-year overall survival (A) or 10-year progression-free survival (B).**

tAUC, the time-dependent area under the receiver operator curve; L/S, long-to-short axis.


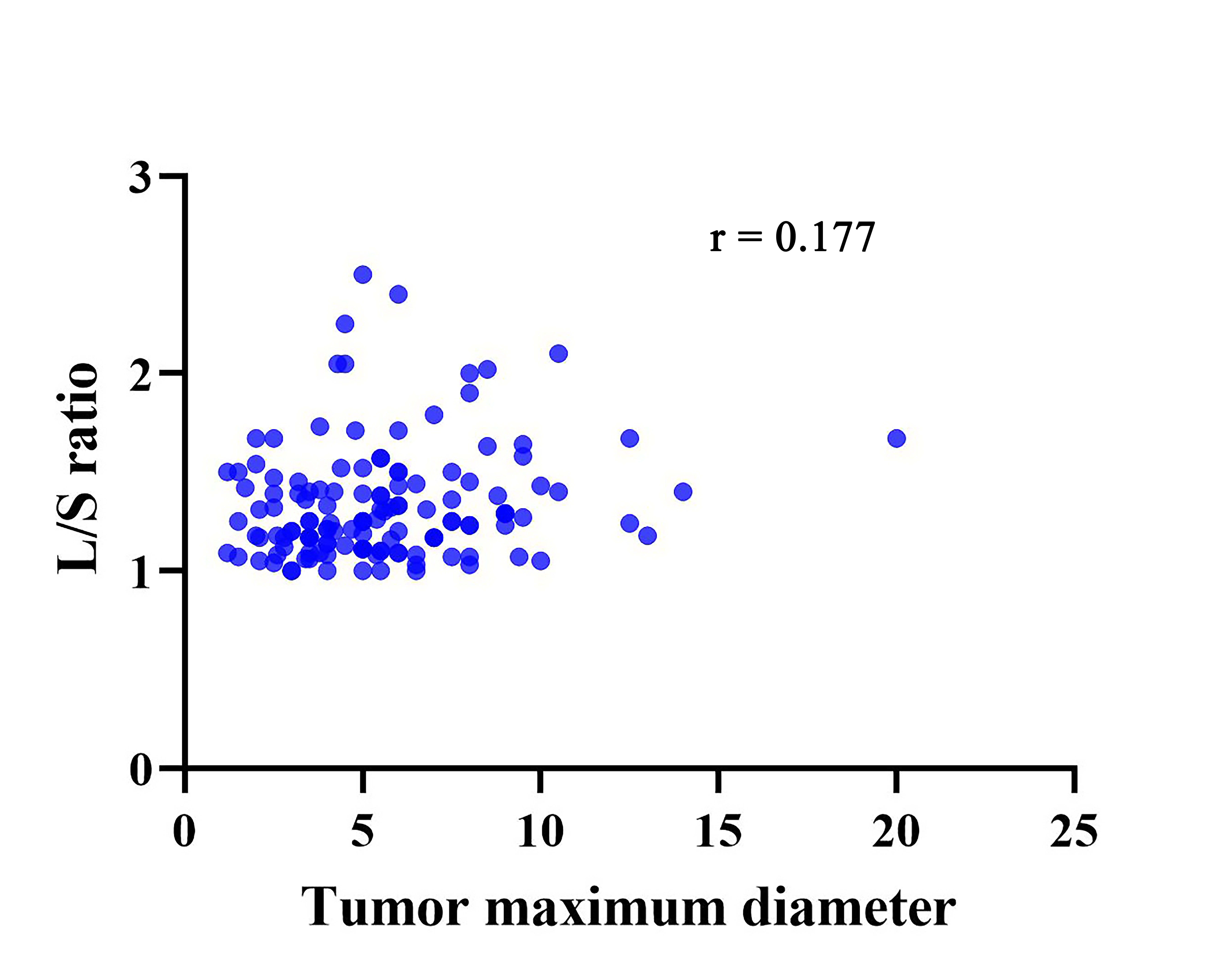


**FIGURE E5. The distribution of tumor maximum diameter and L/S ratio.**

The Pearson’s correlation coefficient between tumor maximum diameter and L/S ratio was 0.177.

L/S, long-to-short axis.

**FIGURE E6. Histopathology of TETs with high- and low- L/S ratio**

**(A)** The high L/S ratio TETs show no fibrous septum (HE × 10).

**(B)** The low L/S ratio TETs appears as more fibrous septum (HE × 10).

TETs, thymic epithelial tumors; L/S ratio, long-to-short axis ratio; HE, hematoxylin-eosin.
